# Novel *RB1* and *MET* Gene Mutations in a Case with Bilateral Retinoblastoma Followed by Multiple Metastatic Osteosarcoma

**DOI:** 10.3390/diagnostics11010028

**Published:** 2020-12-25

**Authors:** Attila Mokánszki, Yi-Che Chang Chien, János András Mótyán, Péter Juhász, Emese Sarolta Bádon, László Madar, István Szegedi, Csongor Kiss, Gábor Méhes

**Affiliations:** 1 Department of Pathology, Faculty of Medicine, University of Debrecen, H-4032 Debrecen, Hungary; dr.changchien.yiche@med.unideb.hu (Y.-C.C.C.), peter.juhasz@med.unideb.hu (P.J.), badon.emese.sarolta@med.unideb.hu (E.S.B.), gabor.mehes@med.unideb.hu (G.M.); 2 Department of Biochemistry and Molecular Biology, Faculty of Medicine, University of Debrecen, H-4032 Debrecen, Hungary; motyan.janos@med.unideb.hu; 3 Doctoral School of Molecular Cell and Immune Biology, University of Debrecen, H-4032 Debrecen, Hungary; mlaszlo0911@gmail.com; 4 Division of Clinical Genetics, Department of Laboratory Medicine, Faculty of Medicine University of Debrecen, H-4032 Debrecen, Hungary; 5 Department of Pediatrics, Faculty of Medicine, University of Debrecen, H-4032 Debrecen, Hungary; iszegedi@med.unideb.hu (I.S.), kisscs@med.unideb.hu (C.K.)

**Keywords:** retinoblastoma, osteosarcoma, *RB1* gene, *MET* gene, somatic mutations, next-generation sequencing (NGS)

## Abstract

Retinoblastoma (Rb) is a malignant tumor of the developing retina that affects children before the age of five years in association with inherited or early germline mutations of the *RB1* gene. The genetic predisposition is also a driver for other primary malignancies, which have become the leading cause of death in retinoblastoma survivors. Other malignancies can occur as a consequence of radiotherapy. We describe a patient with retinoblastoma in which we detected a novel *RB1* c.2548C > T, p.(Gln850Ter) and a synchronous *MET* c.3029C > T, p.(Thr1010Ile) mutation as well. After presenting with bilateral retinoblastoma, the patient developed at least four different manifestations of two independent osteosarcomas. Our goal was to identify all germline and somatic genetic alterations in available tissue samples from different time periods and to reconstruct their clonal relations using next generation sequencing (NGS). We also used structural and functional prediction of the mutant RB and MET proteins to find interactions between the defected proteins with potential causative role in the development of this unique form of retinoblastoma. Both histopathology and NGS findings supported the independent nature of a chondroblastic osteosarcoma of the irradiated facial bone followed by an osteoblastic sarcoma of the leg (tibia).

## 1. Introduction

Retinoblastoma (Rb, OMIM#180200) is a malignant tumor of the developing retina that affects children before the age of five years with an estimated incidence between 1 in 16,000 and 1 in 18,000 live births [1]. Rb occurs in both heritable (25–30%) and nonheritable (70–75%) forms. A heritable form is defined by the presence of a germline heterozygotic variant in the *RB1* gene (Genbank accession number L11910.1; NCBI RefSeq NM_000321.2), which is followed by a second somatic hit in the developing retina. As a result, tumors affecting either one (unilateral) or both (bilateral) eyes may develop. In the nonheritable form, both mutations occur in somatic cells, usually leading to unilateral malignancy [2]. In addition to the highly malignant early onset Rb, the risk of developing second cancers, e.g., osteosarcomas, other soft-tissue sarcomas and rarely melanomas, is increased. Molecular diagnostics is required to clear heredity status and to deliver the best options for the management of the disease [3,4]. Due to the genetic predisposition, second malignant neoplasias (SMN) may arise spontaneously or following radiotherapy. SMNs represent the leading cause of death in Rb survivors. Osteosarcomas in retinoblastoma patients occurred 1.2 years earlier, and the latency period between radiotherapy and osteosarcoma onset was 1.3 years shorter inside the radiation field than outside it [5].

The *RB1* gene shows a wide spectrum of mutations, including single nucleotide variants (SNVs), small insertions/deletions (indels), and large deletions/duplications. These mutations are distributed throughout the entire length of the gene, spanning over 27 exons, and no hotspots have been reported [6]. New advances in molecular genetic testing, and especially next-generation sequencing (NGS), allow the comprehensive demonstration of all SNVs and large aberrations throughout the full length of the gene. Pathogenic variants in both alleles of the *RB1* gene are related to the development of this neoplasm in the large majority of the cases. *RB1* gene aberrations are missing in rare Rb cases, indicating to oncogenic interactions between different signal transduction pathways [7].

The aims of our study were (i) to identify independent de novo second hits in a patient with bilateral Rb of nonparental origin; (ii) to exclude parental carrier status; (iii) to characterize histological and genetic features of samples originating from the two Rb and the four anatomically distinct osteosarcoma tumors; (iv) to identify genetic differences between the irradiation-related orbitofacial and the non-irradiation-related de novo osteosarcoma of the lower extremity; (v) to predict structure and function of the proteins encoded by the mutant genes, as reconstructed based on DNA sequencing; and, finally, (vi) to identify potential interactions between defected proteins using prediction analysis. For this purpose, histology, including immunohistochemistry (IHC) and NGS solid tumor gene panel (Illumina MiSeq platform) analysis, was performed using samples from both enucleated eyes and from the four available osteosarcoma tissues. Autopsy sample of the skin was taken as normal non-neoplastic control tissue. In addition, in silico prediction methods were applied to analyze the secondary structure and functionality of detected germline variants and to predict protein–protein interactions. To exclude potential parental origin, conventional Sanger sequencing was used.

## 2. Materials and Methods

### 2.1. Patients Samples

Altogether, seven formaldehyde-fixed paraffin-embedded tissue (FFPE) samples were tested from the patient diagnosed and treated with Rb/osteosarcoma between 2010 (at first admission due to Rb) and 2019 (at the time of death of the patient) at the Department of Pediatrics, University of Debrecen (Table 1). Peripheral blood samples from both parents were collected for analyzing their carrier status. Sampling was agreed and supported by a written consent from both sides. All protocols have been approved by the author’s respective Institutional Review Board for human subjects (IRB reference number: 60355/2016/EKU) and conducted in accordance with the Helsinki Declaration.

### 2.2. Histology and Immunohistochemistry

Hematoxylin and eosin (H&E) stained slides were carefully evaluated by two independent pathologists, and appropriate tumor samples were selected for DNA isolation with a tumor percentage >20%. In samples where lower tumor ratio was detected, microdissection was performed. IHCs of neuron-specific enolase (NSE, BBS/NC/VI-H14 clone, 1:800 dilution, Agilent Technologies, Santa Clara, CA, USA) and synaptophysin (27G12 clone, 1:100 dilution, Leica Biosystems, Wetzlar, Germany) were performed to confirm Rb diagnosis. Anti-RB monoclonal antibody (1F8 (RB1) clone, 1:200 dilution, Invitrogen, Carlsbad, CA, USA) was used on all samples of the patient and on a control colorectal adenocarcinoma sample.

### 2.3. DNA Isolation

DNA isolation from peripheral blood was performed using QIAamp DNA Mini Kit (Qiagen, Hilden, Germany). Genomic DNA was extracted from FFPE tissues using the QIAamp DNA FFPE Tissue Kit (Qiagen, Hilden, Germany). The isolations were carried out according to the manufacturer’s standard protocol, and the DNA was eluted in 50 µL elution buffer. The DNA concentration was measured in the Qubit dsDNA HS Assay Kit using a Qubit 4.0 Fluorometer (Thermo Fisher Scientific, Waltham, MA, USA).

### 2.4. Sanger Sequencing

*RB1* mutation testing (exon 25) was performed using conventional Sanger sequencing on all DNA samples originating from FFPE blocks of the patient and on the peripheral blood DNA samples of their parents. The purified PCR products were sequenced using both forward and reverse primers (which were used for the PCR amplification) using the BigDye Terminator v3.1 Cycle Sequencing kit. The samples were analyzed on the ABI PRISM 310 Genetic Analyzer (Thermo Fisher Scientific, Waltham, MA, USA).

### 2.5. Next-Generation Sequencing

The amount of amplifiable DNA (ng) was calculated according to the Archer PreSeq DNA Calculator Assay Protocol (Archer DX, Boulder, CO, USA). After fragmentation of the genomic DNA, libraries were created by the Archer VariantPlex Solid Tumor Kit covering 67 genes (Archer DX, Boulder, CO, USA). The KAPA Universal Library Quantification Kit (Kapa Biosystems, Roche, Basel, Switzerland) was used for the final quantification of the libraries.

The MiSeq System (MiSeq Reagent kit v3 600 cycles, Illumina, San Diego, CA, USA) was used for sequencing. The libraries (final concentration of 4 nM, pooled by equal molarity) were denatured by adding 0.2 nM NaOH and diluted to 40 pM with hybridization buffer from Illumina (San Diego, CA, USA). The final loading concentration was 8 pM libraries and 1% PhiX. Sequencing was conducted according to the MiSeq instruction manual. Captured libraries were sequenced in a multiplexed fashion with paired end run to obtain 2x150 bp reads with at least 250X depth of coverage. The trimmed fastq files were generated using MiSeq reporter (Illumina, San Diego, CA, USA).

Raw sequence data were analyzed with Archer analysis software (version 6.2.; Archer DX, Boulder, CO, USA) for the presence of single-nucleotide variants (SNVs) as well as insertions and deletions (“indels”). For the alignment, the human reference genome GRCh37 (equivalent UCSC version hg19) was built. Molecular barcode (MBC) adapters were used to count unique molecules and characterized sequencer noise, revealing mutations below standard NGS-based detection thresholds. The sequence quality for each sample was assessed and the cutoff was set to 5% variant allele fraction. Large insertion/deletion (> 50 bp) and complex structural changes could not be captured by the method. The results were described using the latest version of Human Genome Variation Society nomenclature for either the nucleotide or protein level. Individual gene variants were cross-checked in the COSMIC (Catalogue of Somatic Mutations in Cancer) and ClinVar databases for clinical relevance. We used the gnomAD v.2.1.1 population database to compare the significance of each gene alteration included in our Archer NGS analysis system.

### 2.6. Protein in Silico Methods

Protein information for the RB protein (P06400, RB_HUMAN) and for the hepatocyte growth factor receptor MET (P08581, MET_HUMAN) were obtained from the UniProt database and from RCSB Protein Data Bank. The GOR method (version IV) was used for secondary structure prediction [8]. Disorder prediction was performed by the IUPred2A web server [9]. Eukaryotic linear motifs (ELM) were identified in the proteins by using the ELM database [10]. Stability changes upon point mutations were predicted by the I-Mutant 2.0 web server based on protein sequence, using default parameters [11]. Protein–protein interaction data were obtained from the BioGRID [12] and STRING databases [13].

## 3. Results

### 3.1. Clinical Presentation and Management

A one-year-old male with a suspicion of Rb was referred to the Department of Pediatrics, University of Debrecen. Given the bilateral, large extension of the tumors not suitable for local therapy, systemic chemotherapy was immediately introduced. Two cyclophosphamide/vincristine blocks, advised for infant neuroblastoma, were applied with partial response. Thus, therapy was continued with three more intensive carboplatin/etoposide/vincristine blocks, but without further tumor regression, so three additional VEC (vincristine/etoposide/cyclophosphamide) blocks were introduced that resulted in significant tumor regression, making local therapy possible. Before the start of the local intervention, two additional VEC cycles were applied with decreased (60%) doses taking the tolerance of the patient in consideration. Local brachytherapy (Ruthenium-106 applicator) was applied on the right side, and cryotherapy on the left side. Five months later a progression was observed on the right side that extended the borders of local control. As a bridging therapy, a VEC cycle with additional cisplatin was applied. Then, the patient received 50 Gy external beam radiotherapy to both sides. Repeated progression occurred five months later; thus, enucleation was necessary on the right, and then, unfortunately, on the left side three months apart (Sample 1 (S1) and 2 (S2)). Telemetric radiotherapy followed enucleation on both sides in 50 Gy doses. The proband, at the age of 10 years, presented with osteosarcoma of the left orbit that was surgically resected (S3). The EURAMOS1 protocol, rather than the EURO EWING99-VIDE protocol, was used for chemotherapy. One year later. Osteosarcoma also presented on the left tibia, and 17 cm of its proximal region was resected (S4). The resected bone was irradiated using 100 Gy extracorporeal radiation, and EURAMOS1/COSS chemotherapy was applied. At the age of 12 years, left femur and multiple pulmonary metastases were diagnosed. Up to the upper third, the left femur was amputated, and osteosarcoma was proved by histological examination (S5). Due to the bad general condition of the child, the pulmonary metastasis was considered inoperable. Three months later, the proband died due to respiratory complications. Progressive metastases of both sides of the lungs were demonstrated as cause of death during autopsy. Postmortem sampling from the pulmonary metastases (S6) and from the intact skin (sample S7, for nontumor control purposes) was conducted. Samples provided for the comparative study and related histological diagnoses are summarized in Table 1.

### 3.2. Histological Examinations

Sections from the left and right ocular tumors showed a picture of s small round cell tumor with high mitotic activity and tumor necrosis. The tumor cells possessed neuroendocrine immunophenotype (NSE and synaptophysin positivity). Diagnosis of Rb was established.

**Figure 1:**
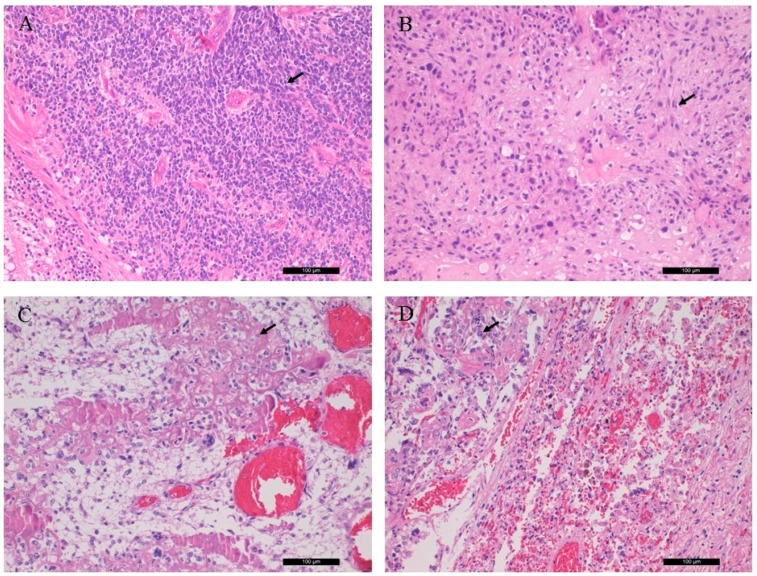
Conventional histological (H&E) characteristics of patient tumor samples. (**A**) Rb of the right eye bulb, (**B**) chondroblastic osteosarcoma of the left orbital bone, (**C**) osteoblastic osteosarcoma from the left tibia, (**D**) osteoblastic osteosarcoma, pulmonary metastasis. Arrows indicate retinoblasts, as well as chondroblastic and osteoblastic propagation (magnification: ×20).

The sections from the left orbital and left tibia/femoral tumors revealed chondroblastic and osteoblastic osteosarcoma, respectively, with high grade, irregular tumor cell proliferation and osteoid production. The former sample also showed excess amount of malignant cartilaginous component. The lung tumor showed classical osteoblastic osteosarcoma, indicating the metastatic nature from the lower leg. Key histological features are shown in Figure 1.

### 3.3. RB1 and MET Mutation Analysis

In all samples of the patient, the same c.2548C > T, p.(Gln850Ter) mutation of the *RB1* gene was detected in a heterozygous form (variant allele frequency (VAF): 50 ± 10%). In addition, the c.3029C > T, p.(Thr1010Ile)/c.2975C > T, p.(Thr992Ile) variant of the *MET* gene was identified throughout the samples (VAF range: 27–50%). Targeted Sanger sequencing of the parental DNA isolated from the peripheral blood did not detect the variants in question.

### 3.4. Immunohistochemistry of the RB Protein

**Figure 2:**
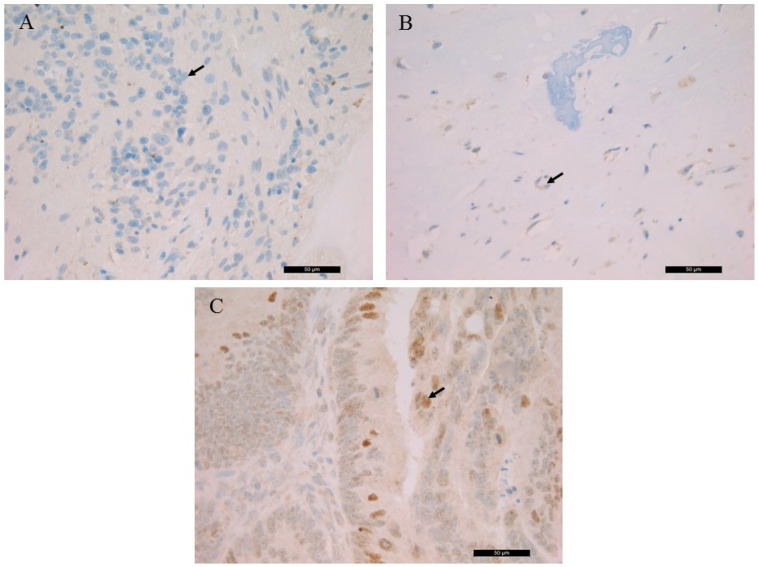
Immunohistochemistry of the RB protein. (**A**) retinoblastoma (S1), (**B**) left orbital bone (S3), (**C**) colorectal adenocarcinoma control. Arrows indicate the immunohistochemistry (IHC)-negative cell nuclei of retinoblastic/chondroblastic tumors and the positive nuclear staining in adenocarcinoma cells (magnification: ×40).

The detected germline *RB1* gene mutation suggested the generation of a truncated RB protein following translation. The histological localization and intracellular distribution of the aberrant protein was analyzed using an anti-RB monoclonal antibody on all samples and on a known positive control (colorectal adenocarcinoma). While the nuclear localization of the RB protein was shown in control slides. it was not detected in the patient samples. RB IHC of the S1, the S3 and the control is presented in Figure 2.

### 3.5. Somatic Gene Mutations

The molecular genetic results of the samples originating from the different tumor type are presented in Table 2.

In the Rb sample from the right eye bulb (S1), *CTNNB1* and *EZH2* mutations were identified opposite to the S2 (Rb from the left eye bulb) tumor, where *ALK*, *APC* and *CDH1* variants were detected. *EZH2* aberration emerged in the all tumor samples, but not in the nontumor control; therefore, this was considered as a tumor-specific somatic gene variant. In the chondroblastic osteosarcoma sample (S3), no other somatic mutations were detected. In contrast, all osteoblastic osteosarcoma samples (S4–S6) featured the same *TP53* pathogenic variant with high VAF (38.4–69.0%). Additional variants of the genes *ERBB*, *HRAS* and *SMAD4* were identified at the level of the individual samples.

### 3.6. In Silico Analysis of the RB and Hepatocyte Growth Factor Receptor (MET) Mutant Proteins

**Figure 3:**
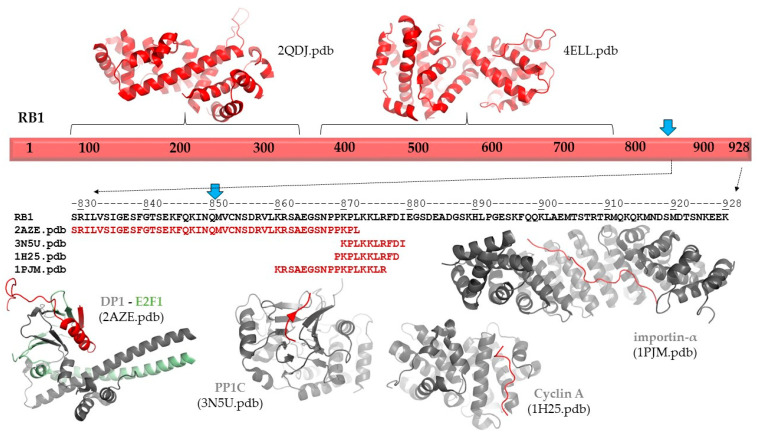
Structure and interactions of RB1. Schematic representation of the full-length RB1 protein is shown. The structures of the N-terminal and central regions are represented, and the protein–protein interactions of the C-terminal region are also shown based on structural data. The sequence of the C-terminal of region of RB1 is shown by black color. Blue arrows show the position of the p.Gln850Ter mutation. The sequences that are involved in protein–protein interactions are shown for each coordinate file; these regions are also highlighted in red in the complex structures.

The p.(Gln850Ter) mutation of the RB protein causes a truncation of the full-length protein (1–928) by 78 residues (Figure 3). The deletion of the C-terminal region (851–928 residues) was predicted to cause no alteration of the secondary structural arrangement, and neither a local nor long-distance effect was predicted for the truncated protein. Disorder prediction also showed that the truncation of the protein does not cause significant changes in pathogenicity.

Neither secondary structure nor disorder predictions showed any adverse effects of the deletion on protein structure. Therefore, the consequence of the truncation can be associated with the loss of functional regions. The functional importance of the C-terminal region in RB protein is implied by the presence of ELMs, including a bipartite nuclear localization signal (860–876). Consequently, the loss of this signal sequence may result in impaired nuclear localization of the mutant/truncated protein, while the wild-type protein naturally enters the nucleus. The structure and possible interactions of the RB1 protein are presented in Figure 3. The following coordinate files were used to prepare the figure: 2QDJ.pdb [14], 4ELL.pdb [15], 2AZE.pdb [16], 3N5U.pdb [17], 1H25.pdb [18], and 1PJM [19].

The p.(Thr1010Ile) mutation of the MET protein causes a nonsynonymous alteration of a polar threonine to a hydrophobic isoleucine residue. The possible effects of this mutation were predicted by multiple algorithms which showed no alteration of the secondary structural arrangement at or in the proximity of the mutated residue. The disorder propensities predicted for the mutant were highly similar to those obtained for the wild type. In agreement with this, the sequence-based prediction of stability changes also implied neutral nature of the mutation (−0.37 kcal/mol). We predicted no significant increase or decrease in the free energy change value upon p.(Thr992Ile) mutation. Similar to the RB protein, no local or global disturbances of the structure could be predicted; rather, functional changes of the residue in 992^th^ position may be responsible for the phenotypic effects.

### 3.7. Interaction Analysis between the RB and MET Protein

Based on data available in the BioGRID and STRING databases, no direct interaction between RB1 and MET proteins could be established. This is supported by the different subcellular localizations of the wild-type proteins, as RB1 and MET act in diverse subcellular compartments (in the nucleoplasm and in the cytoplasm, respectively).

However, RB1 and MET share multiple interaction partners. Based on the BioGRID database, common interacting partners include CDK4, CDK6, GRB2, MYC, and RAF1 proteins. Thus, simultaneous functional changes of RB1 and MET may basically transactivate the existing signaling networks.

## 4. Discussion

The c.2548C > T, p.(Gln850Ter) *RB1* germline gene variant is registered in the COSMIC databases, but no relation to Rb has been documented to date. Therefore, this was the first time to describe the variant in Rb. Germline loss of function *RB1* gene mutations are known to be causative in Rb [6] and are associated with increased risk of osteosarcoma development [20]. The predisposition to sarcomas has been attributed to genetic susceptibility due to inactivation of the *RB1* gene as well as to the genotoxic effect of radiotherapy applied to treat Rb. Bone and soft-tissue sarcomas in survivors of hereditary Rb occur most frequently within the radiation field in the facial bone (orbit), but they may also occur elsewhere. *RB1* alterations also serve as unfavorable prognostic markers for the clinical classification and management of osteosarcoma patients [21]. 

In agreement with disordered structural variants of the RB protein, the C-terminal region has already been reported to participate in protein–protein interactions. Structural studies revealed interactions of the RB protein with the heterodimer of E2F transcription factor 1 (E2F1) and transcription factor Dp-1 (DP1 (829–874) [18]; with the catalytic subunit of protein phosphatase 1 (PP1c) (870–882) [19]; with the complex of cyclin dependent protein kinase 2 (CDK2) and cyclin A (868–878) [20]; and with mouse importin-α (858–877) [19] (numbers in parentheses show regions of the retinoblastoma-associated proteins that form interactions in the complexes; Figure 3). Additionally, interactions between the RB protein and the complex of cyclin-dependent protein kinase 9 (CDK9) and cyclin T2 were also reported, and this mutagenesis study revealed that the interactions were mediated by the C-terminus of RB protein (835–928) [22]. Among these proteins, only E2F1 has exclusive nuclear localization based on the Human Protein Atlas [23]; the other relevant human proteins are localized in the cytoplasm. Consequently, deletion of the region 851–928 of RB protein most probably influences a series of functionally relevant protein–protein interactions, rather than deletion-induced structural changes promoting directly the pathogenic phenotype.

The diversity of cancers in which *MET* mutations have been identified suggests that the MET protein, activated by mutations, plays an important role in the tumorigenic process in a wide range of cell types. The juxtamembrane domain mutations were shown to attenuate MET receptor ubiquitination and degradation and to prolong MET signaling [24]. There are no eukaryotic linear motifs in the MET protein, which include Thr1010 residues, but based on the PhosphoSitePlus database [25], this residue is known to be phosphorylated. The functional characteristics of the p.(Thr1010Ile) sequence variant has already been reported. Moreover, the transforming nature of this variant was described in a study. The investigation revealed that this variant was present in individuals with or without cancer, and no evidence was found regarding the transformative capacity of the p.(Thr1010Ile) variant [26]. This finding may indicate that the structural integrity of MET has been retained in the mutant. However, structural consequences of the p.(Thr1010Ile) variant have not been investigated to date. In the COSMIC database, this mutation was also described as a germline and somatic form. It proved to be more active than the wild-type MET in the athymic nude mouse tumorigenesis assay, suggesting its potential effect on tumorigenesis [27]. 

In a novel large cohort study, patients with heritable Rb had a significantly increased risk for SMNs, while patients with nonheritable Rb did not. The overall mortality rate was 48% for heritable Rb and 23% for patients with the nonheritable form. The cumulative mortality rate from second cancers at the age of 60 years was 34% among those with heritable Rb and 12% among those with nonheritable Rb. Sarcoma was the most common histological type of malignancy in patients with heritable Rb, and carcinoma was the most common type in nonheritable Rb [28].

Loss of *RB1* gene function has also been found to be associated with increased risk of osteosarcoma metastasis and a poor histological response to chemotherapy as compared with osteosarcoma patients with intact *RB1* function [29]. The majority of bone sarcomas occurred within the radiation field in the head-and-neck region, but up to 40% were diagnosed outside the treatment field, primarily in the lower extremities. Osteosarcoma is most frequently seen in the femur, and osteoblastic osteosarcoma is the most common histological subtype, making up 41–89% of this malignancy [21]. In addition, both chondrosarcoma and Ewing sarcoma have been reported [30]. Sarcomas occurring in the radiation field were diagnosed with a lag time one year shorter than those diagnosed outside the field [5]. In our study, we demonstrated different somatic mutation profiles for osteosarcomas with chondroblastic and osteoblastic phenotypes by the use of a 67 gene solid tumor NGS panel. *TP53* and other gene aberrations were limited to the osteoblastic form of the lower extremity, suggesting an independent evolution from the chondroblastic type originating at the site of irradiation therapy.

The loss or inactivation of *RB1* function results in a significant 1.62-fold increase in mortality rates in patients with osteosarcoma compared with those in patients without this gene aberration [21]. Osteosarcoma is characterized by its high potential to metastasize to the lungs or other bones [31]. Mutations of the *RB1* gene result in its dissociation from E2F and subsequent transcription of multiple genes involved in cell cycle progression, leading to malignant transformation and the progression of osteosarcoma [32]. Metastatic osteosarcoma is typically difficult to control and known to indicate poor prognosis [33].

Because of the expanding number of registered Rb cases [28], the publication of novel rare cases is very important to understand the molecular mechanism of this malignancy. To the best of our knowledge, this is the first report to detect this novel form of *RB1* and synchronous *MET* gene mutation causing nonheritable bilateral retinoblastoma and consequential chondroblastic and osteoblastic osteosarcoma, the latter developing pulmonary metastases. Structural and functional prediction of the germline mutant proteins suggested an indirect parallel involvement in the pathogenesis of this unique series of retinoblastoma-related neoplastic diseases. The results of the 67 gene NGS panel clearly differentiated the histologically identified osteosarcoma types by two different sets of gene variants.

## Figures and Tables

**Table 1:** Sample types and related histological diagnoses of the seven tissue samples used for DNA sequencing. S1–S7: sample 1–7, Rb: retinoblastoma.

Sample	S1	S2	S3	S4	S5	S6	S7
Localization	right eye bulb	left eye bulb	left orbit	left tibia	left femur	lung	skin
Histological diagnosis	Rb	Rb	chondroblastic osteosarcoma	osteoblastic osteosarcoma	osteoblastic osteosarcoma	osteoblastic osteosarcoma	control sample
Time from the Rb diagnosis (months)	25	28	110	125	131	134	134
Estimated tumor ratio (%)	30	5	30	70	60	60	0

**Table 2:** Somatic gene mutations, sequence variants and their frequencies determined by next-generation sequencing (S1–S7: patient’s samples, P1 and P2: parents’ samples). Clinical significance was determined according to the COSMIC database.

Gene Symbol	Gene Name	Nucleotide Change	Amino Acid Change	S1	S2	S3	S4	S5	S6	S7	P1	P2	Clinical Significance
				Variant Allele Frequency (%)									
*ALK*	Anaplastic lymphoma tyrosine kinase	c.3823C > T	p.Arg1275Ter	0	20.3	0	0	0	0	0	0	0	pathogenic
*APC*	Adenomatous polyposis coli	c.7610C > T	p.Ser2537Phe	0	20	0	0	0	0	0	0	0	uncertain
*CDH1*	Cadherin-1	c.1417G > A	p.Glu473Lys	0	20.8	0	0	0	0	0	0	0	pathogenic
*CTNNB1*	Catenin beta-1	c.59C > T	p.Ala20Val	15	0	0	0	0	0	0	0	0	pathogenic
*ERBB4*	Receptor tyrosine-protein kinase erbB-4	c.493G > A	p.Asp165Asn	0	0	0	8.8	0	0	0	0	0	pathogenic
*EZH2*	Enhancer of zeste homolog 2	c.1837-6C > T	splice region	53	48.6	21.4	63.6	61.3	51.6	0	0	0	uncertain
*FOXL2*	Forkhead box protein L2	c.761C > T	p.Ser254Leu	0	15	0	0	0	0	0	0	0	pathogenic
*HRAS*	Transforming protein p21	c.290 + 8C > T	splice region	0	0	0	9.2	0	0	0	0	0	uncertain
*MET*	Hepatocyte growth factor receptor	c.3029C > T	p.Thr1010Ile	49.1	44.4	50	31.5	27	49.2	27.3	0	0	pathogenic
*RB1*	Retinoblastoma protein	c.2548C > T	p.Gln850Ter	44.5	54.6	59	45.3	41.2	50	45.2	0	0	pathogenic
*SMAD4*	SMAD family member 4	c.1487G > A	p.Arg496His	0	0	0	0	0	35.5	0	0	0	pathogenic
*TP53*	Tumor protein p53	c.-29 + 1G > A	splice region	0	0	0	66	69	38.4	0	0	0	pathogenic

## Data Availability

The data presented in this study are available on request from the corresponding author. The data are not publicly available due to protect the rights of parents privacy.
